# Aquaporin-6 is expressed along the rat gastrointestinal tract and upregulated by feeding in the small intestine

**DOI:** 10.1186/1472-6793-9-18

**Published:** 2009-10-07

**Authors:** Umberto Laforenza, Giulia Gastaldi, Mariarosa Polimeni, Simona Tritto, Marisa Tosco, Ulderico Ventura, Manuela F Scaffino, Masato Yasui

**Affiliations:** 1 Department of Physiology, Section of Human Physiology, University of Pavia, Via Forlanini 6, 27100, Pavia, Italy; 2 Department of Experimental Medicine, Section of Normal Human Anatomy, University of Pavia, Via Forlanini 6, 27100, Pavia, Italy; 3 Department of Biomolecular Sciences and Biotechnologies, University of Milan, Via Celoria 26, Milano, Italy; 4 Department of Pharmacology, Keio University School of Medicine, 35 Shinanomachi, Shinjuku-ku, Tokyo, 160-8582, Japan

## Abstract

**Background:**

Several aquaporins (a family of integral membrane proteins) have been recently identified in the mammalian gastrointestinal tract, and their involvement in the movement of fluid and small solutes has been suggested. In this direction we investigated, in some regions of the rat gastrointestinal tract, the presence and localization of aquaporin-6, given its peculiar function as an ion selective channel.

**Results:**

RT-PCR and immunoblotting experiments showed that aquaporin-6 was expressed in all the investigated portions of the rat gastrointestinal tract. The RT-PCR experiments showed that aquaporin-6 transcript was highly expressed in small intestine and rectum, and less in stomach, caecum and colon. In addition, jejunal mRNA expression was specifically stimulated by feeding.

Immunoblotting analysis showed a major band with a molecular weight of about 55 kDa corresponding to the aquaporin-6 protein dimer; this band was stronger in the stomach and large intestine than in the small intestine. Immunoblotting analysis of brush border membrane vesicle preparations showed an intense signal for aquaporin-6 protein.

The results of in situ hybridization experiments demonstrate that aquaporin-6 transcript is present in the isthmus, neck and basal regions of the stomach lining, and throughout the crypt-villus axis in both small and large intestine. In the latter regions, immunohistochemistry revealed strong aquaporin-6 labelling in the apical membrane of the surface epithelial cells, while weak or no labelling was observed in the crypt cells. In the stomach, an intense staining was observed in mucous neck cells and lower signal in principal cells and some parietal cells.

**Conclusion:**

The results indicate that aquaporin-6 is distributed throughout the gastrointestinal tract. Aquaporin-6 localization at the apical pole of the superficial epithelial cells and its upregulation by feeding suggest that it may be involved in movements of water and anions through the epithelium of the villi.

## Background

The gastrointestinal tract moves a large amount of fluid, quantitatively second only to that of the kidney. In man, about 9 L/day of water (deriving from diet and digestive juices) are absorbed, and about 1-2 L/day are secreted with enteric juice [1-3]. Like the kidney, the intestine can also contribute to water homeostasis maintenance by modulating water (and electrolytes) absorption and secretion [4]. Transporting epithelia offer two pathways for water and solute flows: 1) the paracellular route, consisting of junctional complexes and lateral intercellular spaces; 2) the transcellular route, consisting the apical and basolateral cell membranes [4]. These routes are not completely independent, since the lateral intercellular spaces may provide a compartment in which the transport pathways may communicate [5]. Transcellular water transport may occur by several mechanisms but, in the last decade, a special attention has been paid to the role of AQPs, which allow rapid and substantial bi-directional movement of fluid [2,3,6]. Indeed, eight different AQPs have been identified and partially characterized in the gastrointestinal tract of mammals [2,3,7-11].

Aquaporin-6 (AQP6) was first identified in the kidney of the rat (original name WCH3; [12,13] and human (original name hKID; [14]). In the rat, AQP6 gene localizes to chromosome 7 locus 7q36, where it is composed of four exons and three introns. The predicted amino acid sequence contained one consensus site for N-linked glycosylation at Asn-134 and one for phosphorylation at Ser-197 [13]. Expression of AQP6 in *Xenopus laevis *oocytes shows low water permeability (*Pf*) [14,15] which rapidly increases up to ten fold with Hg^2+ ^treatment, unlike other AQPs [15]. Acidic pH (lower than 5.5) also increases the *Pf *of AQP6 oocytes [15]. This activation of AQP6 water permeability is accompanied by an increased ion conductance with the following halide permeability sequence: NO_3_^-^>I^-^>>Br^-^>Cl^-^>>SO_4_^2- ^[15-17]. Thus, differently from other mammalian orthologs AQPs, AQP6 seems to function primarily as an anion-selective channel rather than as a water channel. Moreover, a careful analysis of AQP sequences alignment and mutation experiments demonstrated that the single substitution of the Asn-60 with a Gly residue eliminates the anion permeability and greatly enhances the osmotic water permeability [18]. Whereas most known AQPs are localized on the plasma membrane (except AQP11 [17]), AQP6 in the kidney is localized almost solely in intracellular vesicles (only 1.2% is associated with plasma membrane) [15,19]. Immunogold studies revealed that AQP6 is colocalized with H^+^-ATPase in intracellular vesicles of the intercalated cells of renal collecting ducts [15,19]. Recently, AQP6 was also found in the cerebellum, close to the tight junctions, in secretory granule membranes of parotid acinar cells, and in the apical portion of inner ear supporting cells [20-22], but its expression in the gastrointestinal tissue has been not investigated yet.

In the present study, we investigate whether AQP6 is expressed in several regions of the rat gastrointestinal tract and then define the localization of AQP6 protein, from stomach to rectum, under the hypothesis that it could be involved in physiological mechanisms of fluid movement, acid base regulation and/or anion transport. The expression of AQP6 mRNA and protein in different portions of the rat gastrointestinal tract was studied by RT-PCR and immunoblotting, and the cellular and subcellular localization of mRNA and protein by *in situ *hybridization and immunohistochemistry. Subsequently, the small intestine expression of AQP3, 4, 6, 7 and 8 mRNA was investigated in relation to feeding. This study provides evidence for the expression of AQP6 at the apical side of superficial cells of both small and large intestine and to a minor extent in gastric principal and parietal cells. Moreover, the upregulation of AQP6 by feeding in the small intestine suggests its direct involvement in the absorption of water and anions.

## Results

### RT-PCR analysis of AQ6 mRNA expression in rat gastrointestinal tract

**Figure 1:**
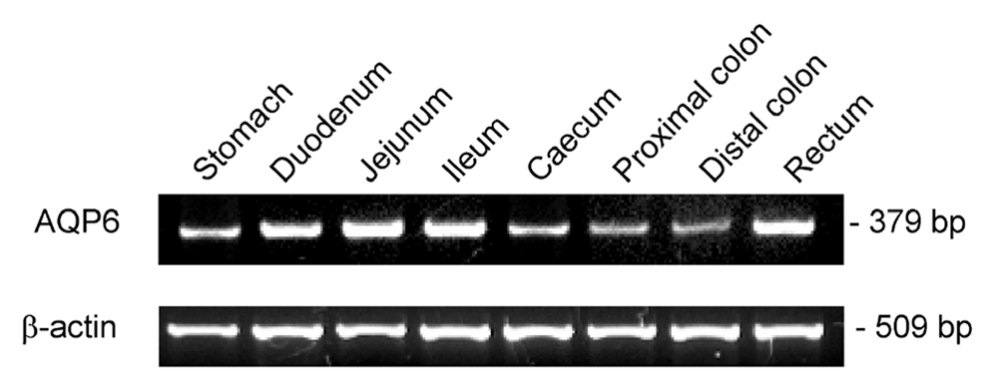
**Distribution of AQP6 mRNA expression in different portions of the rat gastrointestinal tract**. Upper panel, RT-PCR of total RNA from indicated rat samples was performed by using specific primers for AQP6. Lower panel, RT-PCR of β-actin used to normalized the expression of AQP6.

We investigated the expression and distribution of AQP6 mRNA in different portions of rat gastrointestinal tract by semi-quantitative RT-PCR. The expression of AQP6 was normalized using β-actin as internal standard. Single bands of the size expected for cDNA fragments were amplified (379 and 509 bp for AQP6 and β-actin, respectively). The results of agarose gel electrophoresis of representative PCR reaction products are shown in Fig. 1. Negative controls of RT-PCR experiments were always performed by omitting reverse transcriptase (not shown). The specificity of the amplified cDNAs was confirmed by sequencing PCR products. The nucleotide sequence of PCR products was identical to the published sequence for rat AQP6 [GenBank: NM_022181]. AQP6 mRNA was expressed in all the portions of the rat gastrointestinal tract, although expression levels varied between samples (Fig. 1). Densitometric analysis of the bands showed that AQP6 transcript was significantly higher in the small intestine (duodenum, jejunum and ileum) and in the rectum than in the large intestine (caecum, proximal and distal colon). The values (means ± S.E.M.; n = 4), expressed as AQP/β-actin densitometric percentage ratio, were: 15.4 ± 2.7 (stomach); 27.5* ± 1.4 (duodenum); 29.9* ± 3.6 (jejunum); 22.3§. ± 2.4 (ileum); 15.1 ± 1.6 (caecum); 12.7 ± 1.1 (proximal colon); 12.4 ± 1.1 (distal colon); 21.8§ ± 2.6 (rectum). *, P < 0.05 vs. caecum, proximal colon, distal colon and stomach; §, P < 0.05 vs. proximal colon and distal colon (one-way ANOVA followed by Newman-Keuls's *Q *test).

### Immunoblotting analysis of the AQP6 protein in rat gastrointestinal tract

**Figure 2:**
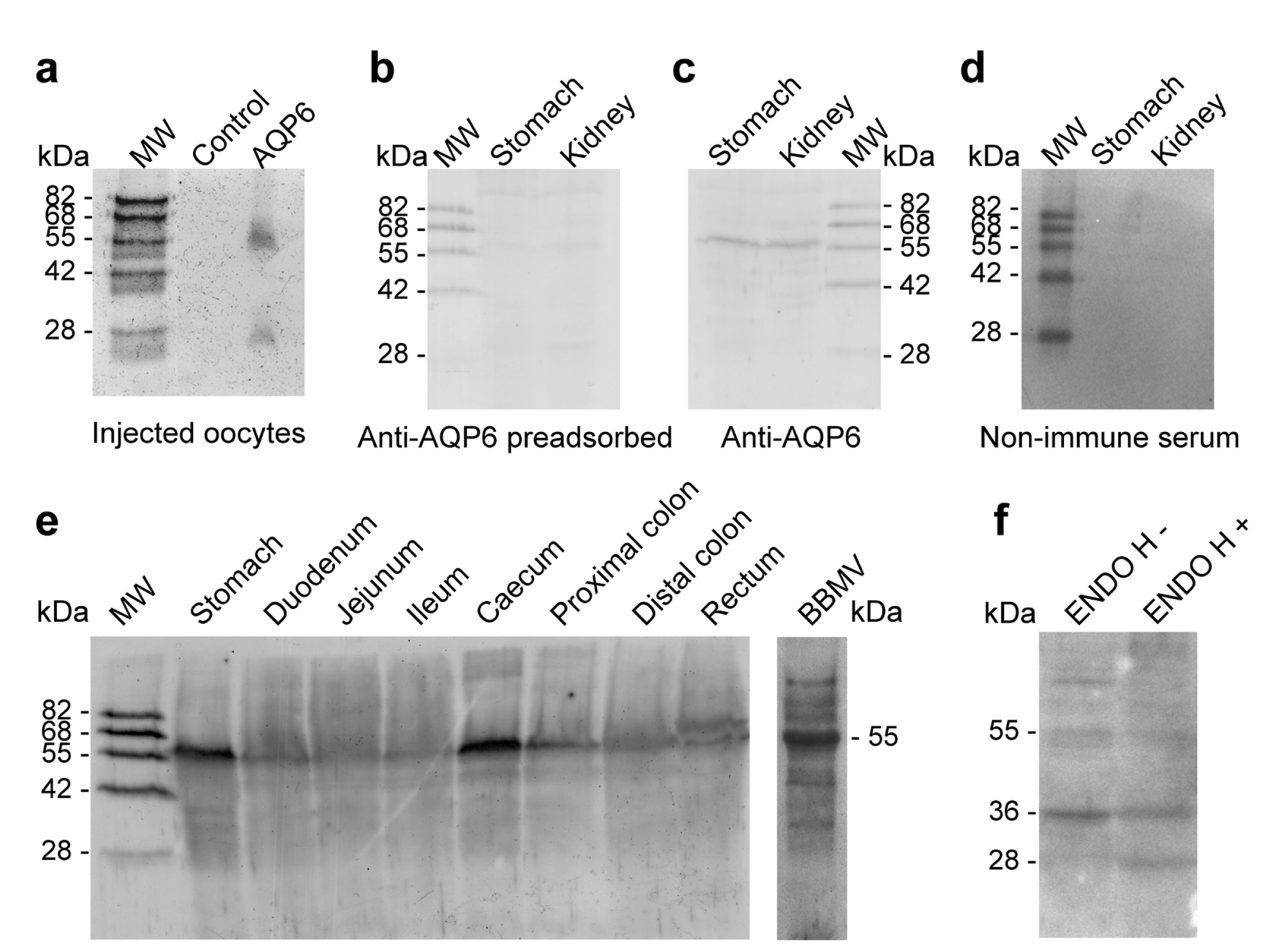
**AQP6 protein expression in rat gastrointestinal tract and brush border membrane vesicles (BBMV)**. **a **Anti-AQP6 immunoblot of membranes isolated from *X. laevis *oocytes injected with AQP6 cRNA and non-injected oocytes (control). Two bands of about 55 and 28 kDa were observed. **b **Immunoblot of membrane fractions from rat stomach and kidney probed with anti-AQP6 preadsorbed with the immunizing peptide, **c**, probed with anti-AQP6 antibody or, **d**, probed with pre-immune rabbit serum. **e **Anti-AQP6 immunoblot of membranes from different portions of the rat gastrointestinal tract (left) and of BBMV from rat small intestine (right). A major band of about 55 kDa was observed (AQP6 dimer). **f **Immunoblot of jejunal homogenates incubated with (ENDO H +) or without (ENDO H -) endoglycosidase Hf. Bands of 55 kDa (AQP6 dimer), 36 kDa (glycosylated form) and 28 kDa (nonglycosylated form) were shown. MW, molecular weight markers.

Total membrane fractions from rat gastrointestinal tract portions and small intestinal brush border membrane vesicles (BBMV) fraction were analyzed by immunoblotting with affinity purified antibodies against rat AQP6. The antibody was preliminary tested on immunoblots of membranes isolated from *X. laevis *oocytes injected with AQP6 cRNA according to Yasui et al. [19]. The results revealed two main bands (28 and 55 kDa) on immunoblots of membranes from AQP6 oocytes (Fig. 2a), consistent with published data [19], while non-injected oocytes were negative (Fig. 2a). Specificity of the reaction was further confirmed by preabsorption experiments performed with total membrane homogenates from rat stomach and kidney (control tissues known to express AQP6) and by incubating the blots with pre-immune rabbit serum. In both cases the AQP6 protein bands (Fig. 2c) were completely ablated (Fig. 2b and 2d). AQP6 immunoblots showed a major band of about 55 kDa (Fig. 2e) in all gastrointestinal tract portions investigated as well as in the kidney (Fig. 2c and 2e). When homogenates were pre-treated at 37°C, bands of 36 and 28 kDa were also observed (see also Fig. 2f and 5b). As resulted from deglycosylation experiments, the 28 and 36 kDa bands represent the non-glycosylated and glycosylated AQP6 monomer forms, respectively (Fig. 2f)(see Additional file 1). The band of 55 kDa may represent an AQP6 dimer, as already reported [19].

AQP6 distribution along the gastrointestinal tract showed highest expression in the stomach followed by caecum, rectum, large and small intestine. To determine whether AQP6 protein is present in intracellular vesicles, as shown in the kidney [19] and/or in plasma membranes, enriched BBMV fractions from rat small intestine were analyzed by immunoblot. An intense signal for AQP6 protein was detected in BBMV preparations, consistent with an apical membrane location of the aquaporin (Fig. 2e).

### In situ hybridization

To study the distribution and localization of AQP6 in the gastrointestinal tract we utilized in situ hybridization and immunohistochemistry techniques in a combined approach.

**Figure 3:**
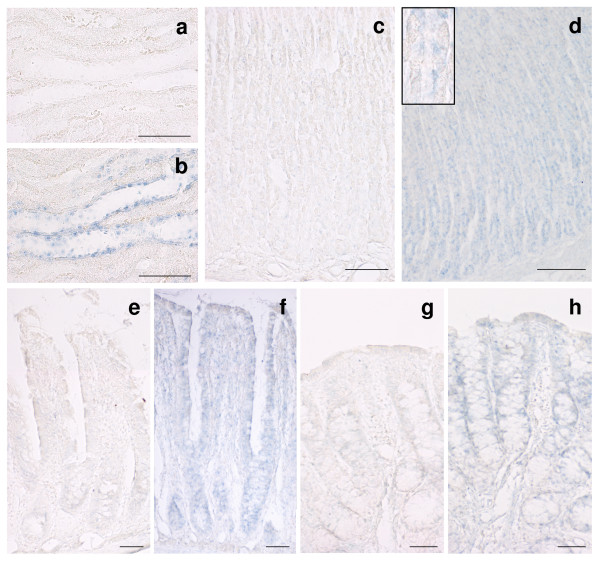
**In situ hybridization analysis of AQP6 mRNA expression**. The probe detects intercalated cells of the collecting ducts in rat kidney (**b**). AQP6 mRNA was detected in cells located in the isthmus, neck and basal region of stomach glands, while pit cells were almost negative (**d**). In jejunum, the probe labelled both crypt and villus epithelial cells (**f**). A similar staining was detected in the colon where labelling is visible along the whole crypt (**h**). No specific signal was detectable in negative control sections from each tissue (**a**, **c**, **e **and **g**). Bars correspond to 100 μm.

Our AQP6 probe was preliminarily tested on kidney sections to verify its specificity and hybridization conditions. As previously reported [23], labelling was observed in type A intercalated cells of the collecting ducts in the inner and outer medulla (Fig. 3b) (see Additional file 2, 3 and 4), and at lower levels in the cortical region, and no labelling was detected in Henle's loop and vessels, as well as in negative control sections (Fig. 3a).

AQP6 transcript was observed in all gastrointestinal segments tested. In the stomach, labelling was found in the isthmus, neck and basal region of glands but almost absent in mucous pit cells (Fig. 3d; inset shows the isthmus-neck region at a higher magnification). In jejunum, AQP6 transcript was detected homogeneously in both crypt and villus epithelial cells (Fig. 3f). The hybridization pattern observed in the colon was similar to jejunum, with labelling distributed in both crypt and surface cells (Fig. 3h). The specificity of hybridization labelling was demonstrated by the low background observed on control sections (Fig. 3a, c, e, g).

### Immunohistochemistry

**Figure 4:**
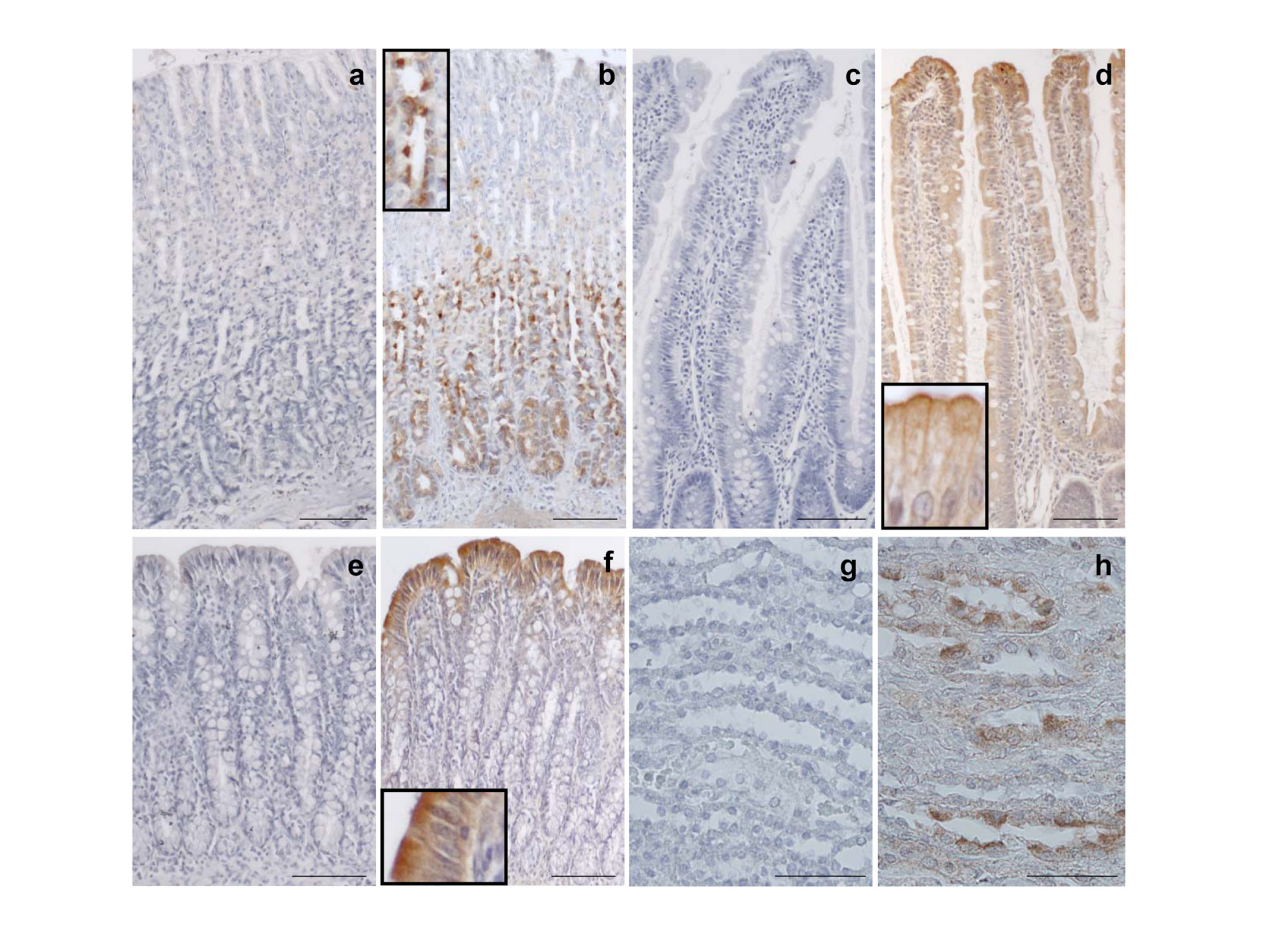
**Immunohistochemical localization of the AQP6 protein in the rat stomach, jejunum and proximal colon**. In the stomach, intense AQP6 staining was observed in the isthmus and neck cells, a lower signal in chief cells and in some parietal cells (**b**, inset). AQP6 immunoreactivity appeared to be confined to intracellular structures. In jejunum, labelling was present in the upper part of the villus and reduced or absent in the crypt (**d**). Intense AQP6 immunoreactivity was observed in the apical part of the epithelial cells (**d**, inset). In the proximal (**f**) part of the colon labelling was present in the surface epithelial cells of the crypt and almost absent in the epithelium at the base of the crypt. Intense AQP6 immunoreactivity was observed in the apical part of the epithelial cells (**f**, inset). Goblet cells were not stained. In the kidney, AQP6 immunoreactivity was observed in the intercalated cells of the collecting duct (**h**). Controls were negative (**a, c, e, g**). Bars correspond to 100 μm. Bars in kidney sections correspond to 50 μm.

The localization of AQP6 was investigated by immunohistochemistry. Preliminarily, the antibody was tested in kidney sections. As previously reported [15,19], an intense labelling was observed the intercalated cells of renal collecting ducts (Fig. 4h). Control sections incubated with pre-immune serum were negative (Fig. 4g). In the stomach labelling was observed in the isthmus, neck and basal regions of glands (Fig. 4b). An intense labelling was also observed in mucous neck cells (MNC, Fig. 4b, inset), which are thought to represent undifferentiated (immature) cells, and lower signal was also observed in chief cells and some parietal cells (Fig. 4b). AQP6 immunoreactivity appeared to be confined to intracellular structures. Controls in which the pre-immune serum was used in place of the primary antibody display no labelling (Fig. 4a). These results agree with those obtained by RT-PCR, in situ hybridization and immunoblotting.

Immunohistochemical analysis of small intestine showed that AQP6 protein was detected in the superficial epithelial cells of the jejunal mucosa (Fig. 4d); similar results were observed also in the duodenum and ileum (not shown).

Differently from results of in situ hybridization experiments, an intense labelling for AQP6 protein was shown in the cells of the upper part of the villus while it was reduced or absent in the crypt (Fig. 4d). At higher magnification, it was possible to note that AQP6 expression was more intense at the brush border membranes of the epithelial cells, even if an intracellular staining was also present (Fig. 4d, inset). This is in agreement with immunoblotting results obtained with BBMV preparation (Fig. 2c). No staining was observed at the basolateral membrane of the epithelial cells (Fig. 4d, inset) nor in goblet cells, resulting in a periodically interrupted immunostaining pattern (Fig. 4d). AQP6 labelling was absent when the primary antibody was substituted by pre-immune serum (Fig. 4c) or when a preadsorbed, affinity purified anti-AQP6 antibody was used (not shown).

In the proximal colon an intense labelling was observed in the superficial epithelial cells; the intensity of AQP6 immunostaining decreased from the surface towards the base of the crypt (Fig. 4f). No labelling was detectable in the intercalated goblet cells (Fig. 4f). At high magnification, the epithelial cells of the proximal colon showed strong AQP-6 immunostaining of the apical membranes but an intracellular signal was also observed (Fig. 4f, inset). Immmunolabelled controls were negative (Fig. 4e).

Similar results were observed also in the caecum, distal colon and rectum (not shown).

### Meal-induced modulation of AQPs expression in rat jejunal mucosa

**Figure 5:**
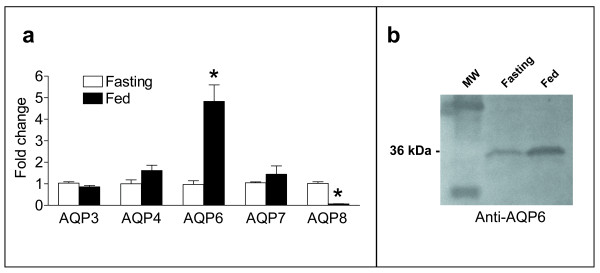
**Aquaporins mRNA and protein expression in rat jejunal mucosa under fasting and fed conditions**. **a **Quantitative real-time RT-PCR of total RNA was performed from jejunal mucosa of rats under fasting (16 h) and fed conditions using specific primers for AQP3, 4, 6, 7, 8. mRNA expression level of AQP6 was significantly higher and that of AQP8 lower in fed condition. *, P < 0.001 vs. fasting (Student's *t *test). MW, molecular weight markers. **b **Immunoblot of jejunal mucosa homogenates from fasting and fed rats.

The expression of AQP3, 4, 6, 7 and 8 mRNA was investigated by quantitative real time RT-PCR (qRT-PCR) in rat jejunal mucosa under fasting and fed conditions. Results showed that feeding significantly increases AQP6 mRNA expression of about 5-fold (Fig. 5a). No significant changes were observed for AQP3, 4, 7, transcripts under these conditions, while AQP8 was dramatically decreased in feeding condition of about 16-fold. AQP6 up-regulation by feeding was also demonstrated at protein level by immunoblotting. As shown in Fig. 5b, AQP6 protein in jejunal mucosa from fed rats was twice as much as that measured in fasting rats.

## Discussion

This study extensively examines the presence, distribution and function of AQP6, at both mRNA and protein levels, in rat gastrointestinal tract. AQP6 was demonstrated, characterized and extensively studied in the α-intercalated cells of the renal collecting duct [16,17,19]. Recent studies demonstrate AQP6 expression in extra renal tissues such as the cerebellum, parotids, and inner ear endorgans [20-22]. The results presented here show for the first time that AQP6 is expressed throughout the gastrointestinal tract (Fig. 1 and 2). RT-PCR experiments reveal that AQP6 transcript is significantly higher in the small intestine and in the rectum than in the other portion of the gastrointestinal tract (Fig. 1). Immunoblotting analysis of crude gastrointestinal tissue homogenates and BBMV preparations show that AQP6 protein expression is higher in the stomach and large intestine than in the small intestine (Fig. 2e). As suggested by immunohistochemistry (Fig. 4d, inset), the small intestine AQP6 immunoreactivity is concentrated in the apical membrane domain (since immunoblotting of BBMV show an increased signal in comparison to the whole homogenate). The specificity of the reaction was tested on immunoblots of membranes isolated from AQP6 oocytes (Fig. 2a), by preadsorbing the antibody with a 20 fold molar excess of immunizing peptide (Fig. 2b) or by incubating the blots with pre-immune-serum (Fig 2d). The AQP6 protein bands were similar to those previously obtained from kidney homogenates [19], although the major band observed here was the 55 kDa (that represents the dimer form) and only occasionally the monomeric form at 28 kDa (Fig. 2c).

The combined analysis of AQP6 mRNA and protein were made to strengthen the results of localization experiments. Except for the stomach, AQP6 mRNA and protein have different patterns of distribution within the gut wall. While mRNA abundance is homogeneous, protein labelling increases from the crypt toward the villus tip. The presence of AQP6 transcript in the crypt cells and lower part of the villus suggests that the gene is transcribed early in the differentiation of epithelial cells, while the protein is present almost exclusively in the mature epithelial cells of the villus tip. Discrepancy between mRNA and protein expression has been observed in the small and large intestine also for AQP3.)[24].

In the stomach, AQP6 mRNA is present in the isthmus, neck and basal region, as observed by in situ hybridization experiments. The results by immunohistochemistry show a strong AQP6 immunolabelling in the isthmus and neck regions and a weak signal in some parietal cells dispersed throughout the glands and in the zymogenic cells at the base of the gland (Fig. 4b).

Based on available data, besides AQP6 only AQP3 and AQP4 are present in the stomach, both at the basolateral side of the cells [2,3]. Nevertheless, the cellular and subcellular localization of AQP6 cannot support its involvement in gastric juice secretion.

Indeed, the intense labelling of MNC appeared located in intracellular vesicles (apical granules) (Fig. 4b, inset). This restrictive localization of AQP6 in the cytoplasm of MNC in close association with small secretory granules was similar to that previously shown for AQP6 in the renal epithelia [19], and for AQP10 in the membrane of the granular vesicles of gastroenteropancreatic endocrine cells of the small intestine [11].

Apart from the well known capacity of the MNC to secrete mucus, the functions of this cell lineage are still obscure. Three possible functions were hypothesized: i) MNC could be undifferentiated (immature) cells, [25,26]; ii) MNC could secrete several peptides involved in both mucosal protection and repairing [27,28]; iii) MNC could contain pepsinogen A and C granules [29]. However, independently from these hypotheses the common characteristic of these cells is the secretory function. Thus, AQP6 expression in the membrane of the MNC secretory granules may be involved in osmotic water movement associated with the exocytotic process. Moreover, the lower AQP6 expression observed in mature chief and parietal cells (Fig. 3b) could indirectly prove the stem cell function of MNC. Indeed, AQP6 may be expressed as an early gene in a developmental stage of the MNC that undergoes gene repression: this stage corresponds to the differentiation process of this cell lineage culminating in the phenotype of the fully functional mature zymogenic and parietal cells. Another important finding of this study is that AQP6 localizes at the apical membrane domain of the superficial epithelial cells of both small and large intestine (Fig. 4d, f) similarly to that observed in the parotid glands. The apical membrane localization of AQP6 has been also confirmed by immunoblotting results obtained with BBMV preparations (Fig. 2e). Thus a role of this water/anion channel in the intestinal absorptive and secretive functions cannot be excluded. This suggestion has been strengthened by the observed increase in AQP6 transcript and protein in fed rat (Fig. 5). Interestingly, of all AQPs studied, AQP6 was the only one upregulated by feeding in the small intestinal mucosa. Higher AQP8 mRNA levels were conversely found in fasting condition. However, further experiments are required to clarify fully the meaning of this finding. Moreover, no modification by feeding was observed in renal mRNA extracts (not shown).

The involvement of a transcellular pathway for water movement across the intestinal epithelium has acquired an ever-growing importance in the last years since several AQPs have been identified in the digestive system of mammals, i.e. AQP1, 3, 4, 5, 7, 8, 9 and 10 [3,7-11,30]. These AQPs have been found in different cells of the intestinal epithelium (enterocytes, goblet cells, endothelial cells and gastroenteropancreatic endocrine cells) and in different cellular membranes (i.e., apical or basolateral). However, the direct AQPs involvement in water transport through apical membranes of the intestinal superficial epithelial cells has been demonstrated only for AQP8 in rat isolated colonocytes [9] and for AQP7 and AQP8 in rat small intestinal BBMV [31].

Recently, AQP10, a new member of the aquaglyceroporin family, was identified in the human small and large intestine [8,32], and, due to its localization on the apical membrane of the absorptive cells, an important role for it in water intestinal absorption was suggested [8]. This hypothesis was successively disproved, since further studies identified two AQP10 isoforms localized in the small intestinal capillary endothelium and in the gastroenteropancrestic endocrine cells [11]. We tested therefore its presence in the rat intestine by RT-PCR but no signal was observed (Laforenza, unpublished data).

We previously proposed a model for transcellular AQP-mediated water movement in the absorptive intestinal cells [10]. In the working model water entered the luminal side through AQP7 and AQP8 and exited at the basolateral side through AQP3 (small intestine: villi, large intestine: surface epithelial cells of the crypts, rectum: surface epithelial cells) and AQP4 (small intestine: crypt cells, large intestine: surface epithelial cells of the crypts) [2,3,9,10,33]. We have now to modify this model by including the data reported here, i.e. the presence of an apical AQP6. However, the unique functional properties of AQP6 do not necessarily suggest its involvement in the apical water transport through the superficial epithelial cells of the small and large intestine.

Even if AQP6 belongs to the aquaporin water channel family, at neutral pH it functions as anion-selective channel [34] and only at pH lower than 5.5 it becomes a functional water channel with maintained ion channel properties. This is interesting in the light of the well know fact that the rat duodenal and jejunal surface have an acidic microclimate (pH 5.2-6.7) that can modulate the intestinal transport of several substances, including weak electrolytes and vitamins [35].

## Conclusion

In conclusion, our results strengthen the role of AQP6 in the homeostatic osmoregulation of the cytoplasm and its organelles, but also, for the first time, suggest a role upregulated by feeding for AQP6 in ion and water absorption in the gastrointestinal tract epithelium.

## Methods

### Animals

Adult Wistar albino rats (350-400 g body weight) reared on a complete standard diet [36] were used. They were housed at the animal facility of the Department of Physiology, Section of Human Physiology, in Pavia, cared for and sacrificed according to the current European legal Animal Practice requirements. Unless otherwise stated gastrointestinal tissues were obtained from fasting rats. Meal-induced modulation of AQPs expression was investigated in jejunal mucosa of rats with unrestricted access to food (Fed) and of fasting rats for at least 16 hours with free access to water (Fasting).

### RNA isolation and RT-PCR

Total RNA was extracted from the gastrointestinal tissues using RNA-Bee™ Isolation Solvent (Tel-Test, Inc., TX, USA). cDNA was synthetized from RNA (1 μg using random hexamers and M-MLV Reverse Transcriptase (Invitrogen, CA, USA). PCR was performed as previously described by Laforenza et al. [9] using specific primers for rat AQP6 (sense, 5'-TTCTGACGCTGCAGTTGGTT-3'; antisense, 5'-TTCTGTGTCCTCTGAGTTCG-3'), AQP3, AQP4, AQP7 and AQP8 [9,10]. Reverse transcription was always performed in the presence or absence (negative control) of the reverse transcriptase enzyme. The RT-PCR reactions were normalized using β-actin as an internal standard [37]. First, the sequences of the AQP bands were checked by using the Big dye terminator cycle sequencing kit (Applied Biosystem, PE, USA). PCR products were separated with agarose gel electrophoresis, stained with ethidium bromide, and acquired with the Image Master VDS (Amersham Biosciences Europe, Italy). Densitometric analysis of the bands was performed by the Total Lab V 1.11 computer program (Amersham) and the results were expressed as a percentage of the AQP/β-actin densitometric ratio. The molecular weight of the PCR products was compared to the DNA molecular weight marker VIII (Roche Molecular Biochemicals, Italy).

### qRT-PCR

qPCR was performed in triplicate using 1 μg cDNA, obtained as above indicated, and specific primers for rat AQP6 (sense, 5'-CGAGAGACCCTTGGAGTCAA-3'; antisense, 5'-AACCAACTGCAGCGTCAGAA-3'; 90 bp PCR product), AQP3, 4, 7 and 8 (see above) MESA GREEN qPCR MasterMix Plus (Eurogentec) was used according to the manufacturer instruction and qPCR performed using Rotor Gene 6000 (Corbett). The conditions were as follows: initial denaturation at 95°C for 5 min; 40 cycles of denaturation at 95°C for 30 s; annealing at 60°C for 30 s, and elongation at 72°C for 40 s. The qRT-PCR reactions were normalized using β-actin as a housekeeping gene. (Rn_Actb_1_SG, QuantiTect Primer Assay QT00193473, Qiagen). The melting curves were generated to detect the melting temperatures of the specific products immediately after the PCR run. The relative mRNA levels were determined by the comparative quantitation method (Corbett) and the results expressed as fold change.

### BBMV preparation

BBMV were isolated from rat small intestine enterocytes by Mg^2+ ^precipitation [38]. BBMV purity was determined by evaluating the degree of enrichment in the marker enzymes saccharase (BBMV marker) and K^+^-stimulated phosphatase (marker for basolateral membranes) as compared to the initial homogenate [39].

The enrichment in saccharase activity was about 14-fold and K^+^-stimulated phosphatase about 0.17-fold.

### Membrane preparation and immunoblotting

The mucosal scrapings from different parts of the gastrointestinal tract and kidneys were homogenized with a Teflon glass Potter-Elvehjem homogenizer (Kontes, Vineland, NJ, USA) in a solution containing: 100 mM NaCl, 1 mM EDTA, 10 mM Tris-HCl, pH 6.8 and 0.1 mg/ml PMSF. After centrifugation at 100000 *g *av for 60 min at 4°C, the pellets were suspended in the homogenization buffer and treated as previously described [40]. Lanes were loaded with 30 μg proteins from kidney or 70 μg of proteins from gastrointestinal tract tissues, subjected to 12.5% SDS-polyacrilamide gel electrophoresis and transferred to the Hybond™ ECL™ nitrocellulose membrane (Amersham) by electroelution. After 3 h blocking with Tris buffered saline (TBS) containing 5% non fat dry milk and 1% Triton X-100 (blocking solution), the membranes were incubated overnight with affinity purified antibody to AQP6 diluted 1:800 in the blocking solution. Antibodies directed against the C-terminal peptide of rat AQP6 with an additional N-terminal cysteine (NH_2_-CKVEKVVDLEPQKKESQTNSEDTEV-COOH) were prepared according to Yasui et al. [19] using standard immunization techniques, and affinity purified using CNBr-activated Sepharose 4B columns coupled to immunizing peptide, according to the recommendations of the manufacturer (Amersham). The purified antibody was preliminary tested by immunoblotting with membranes from 0.3 oocytes injected with AQP6 cRNA [19].

The membranes were washed and incubated for 1 h with peroxidase-conjugated goat antirabbit immunoglobulin G (1:120000 in blocking solution) (Amersham Biosciences Europe, Italy). The bands were detected with ECL™ Advance western blotting detection system (Amersham Biosciences Europe, Italy). The control experiments were performed using the antibody preadsorbed with a 20-fold molar excess of the immunizing peptide and by incubating the blots with pre-immune rabbit serum. In deglycosylation experiments 70 μg of jejunal homogenates were incubated at 37°C in the presence (5000 units) or in the absence of endoglycosidase Hf (New England BioLabs), according to the manufacturer's instructions. Meal-induced modulation of AQP6 protein expression was investigated using jejunal mucosal scrapings from fed and fasting rats. The ChemiBlot™ Molecular Weight Markers were used to accurately estimate the molecular weight and as a positive control for the immunoblot (Chemicon International, Inc., CA, USA).

### In situ hybridization

The AQP6 probe was obtained by subcloning in the pGEM-T Easy vector (Promega, Madison, Wisconsin USA) a 381 bp PCR product amplified from rat kidney cDNA: AQP6 specific primers (sense, 5'-TTCTGACGCTGCAGTTGGTT-3'; antisense, 5'-TTCTGTGTCCTCTGAGTTCG-3') were chosen to amplify a region running from the second half of exon 2, to a few nucleotides before the stop codon located in exon 4. This sequence was chosen on the basis of a BLAST analysis performed to check its specificity as AQP6 probe and the absence of cross reactivity with other rat mRNAs. AQP6 antisense riboprobe was synthesized from the linearized plasmid using T7 RNA polymerase (Invitrogen, Carlsbad California) and digoxigenin-labelled UTP (Boehringer Mannheim, Indianapolis, IN).

In situ hybridization was performed as previously described [41] with minor modification. Briefly, adult tissues were dissected and fixed overnight in 4% paraformaldehyde in PBS, dehydrated and paraffin-embedded. 15 μm sections were collected on 3'-aminopropyltriethoxysilane-coated slides and treated for 10 min. at room temperature with proteinase K (Eurobio, Les Ulis, France). Hybridization was carried out for 16-18 h at 70°C in hybridization buffer containing the antisense AQP6 digoxigenin-riboprobe and 50% formamide, 5× SSC, 1% blocking reagent, 5 mM EDTA, 0.1% tween 20, 0.1% CHAPS, 0.1 mg/ml heparin and 1 mg/ml yeast RNA. After two washes in 2× SSC - 50% formamide, sections were treated for 30 min. at 37°C with 20 μg/ml of RNase A and then washed up to 0.2× SSC at 55°C. Hybridized sections were subsequently blocked 2 h at r.t. with blocking solution (1% Boehringer blocking reagent, 10% goat serum, 0.1% tween 20 in PBS) and then incubated o.n. at 4°C with a polyclonal sheep anti-digoxigenin antibody coniugated to alkaline phosphatase (Boehringer) diluted 1:1000 in PBS. Alkaline phosphatase was visualized by 2-18 h reaction with NBT/BCIP (Boehringer) at r.t. in the dark. Sections were observed with a Nikon Eclipse 80 i light microscope and images were acquired with the Nikon Nis Element F Imaging Software.

### Immunohistochemistry

The rats were anesthetized with halothane and intracardially perfused with acetate-buffered 4% formalin. Gastrointestinal tissues were removed, postfixed for 1 h, and processed into paraffin. Serial paraffin sections (5 μm) were rehydrated and treated as previously described [9]. Control experiments were performed simultaneously using antibodies preadsorbed with an immunizing peptide, omitting the primary antibody or incubating with pre-immune rabbit serum.

The immunostained slides were examined by light microscopy using an Olympus BX41 and the digital images acquired with the Nikon DS-Fi1 digital camera using Nis Element F Imaging Software.

### Protein content

The protein content was determined with the Lowry et al. [42] method using bovine serum albumin as standard.

### Statistics

All data were expressed as means ± S.E.M. The significance of the differences of the means was evaluated with Student's *t *test using GraphPad Prism 4.00, 2003.

RT-PCR gels, immunoblots and micrographs in the figures are representative of at least four separate experiments.

## Authors' contributions

UL designed the study, performed the experiments, analyzed data, and wrote the manuscript. GG and UV analyzed and discussed the results. MP performed the AQP6 probe cloning and the in situ hybridization experiments, analyzed these results and wrote the related text. ST performed the immunoblotting experiments and was involved in revising the manuscript. MFS performed mRNA extraction and the RT-PCR experiments. MT and MY were involved in the analysis of the data and the discussion of the results. All authors read and approved the final manuscript.

## Supplementary Material

Additional file 1 **N-glycosylation of AQP6**. The results of densitometric analysis of immunoblot showed that ENDO H digestion of jejunal membrane proteins decreases both trimer and dimer bands. Click here for file

Additional file 2 **In situ hybridization analysis of AQP6 mRNA expression in rat kidney**. The probe detects intercalated cells of the collecting ducts in rat kidney. Click here for file

Additional file 3 **In situ hybridization analysis of AQP6 mRNA expression in rat kidney**. The probe detects intercalated cells of the collecting ducts in rat kidney. Click here for file

Additional file 4 **In situ hybridization analysis of AQP6 mRNA expression in rat kidney**. The probe detects intercalated cells of the collecting ducts in rat kidney. Click here for file
