# Supplementary figures and images for: Aquaporin-6 is expressed along the rat gastrointestinal tract and upregulated by feeding in the small intestine
Source: BMC Physiol. 2009 Oct 7;9:18. doi: 10.1186/1472-6793-9-18 (PMC2765416; doi:10.1186/1472-6793-9-18)

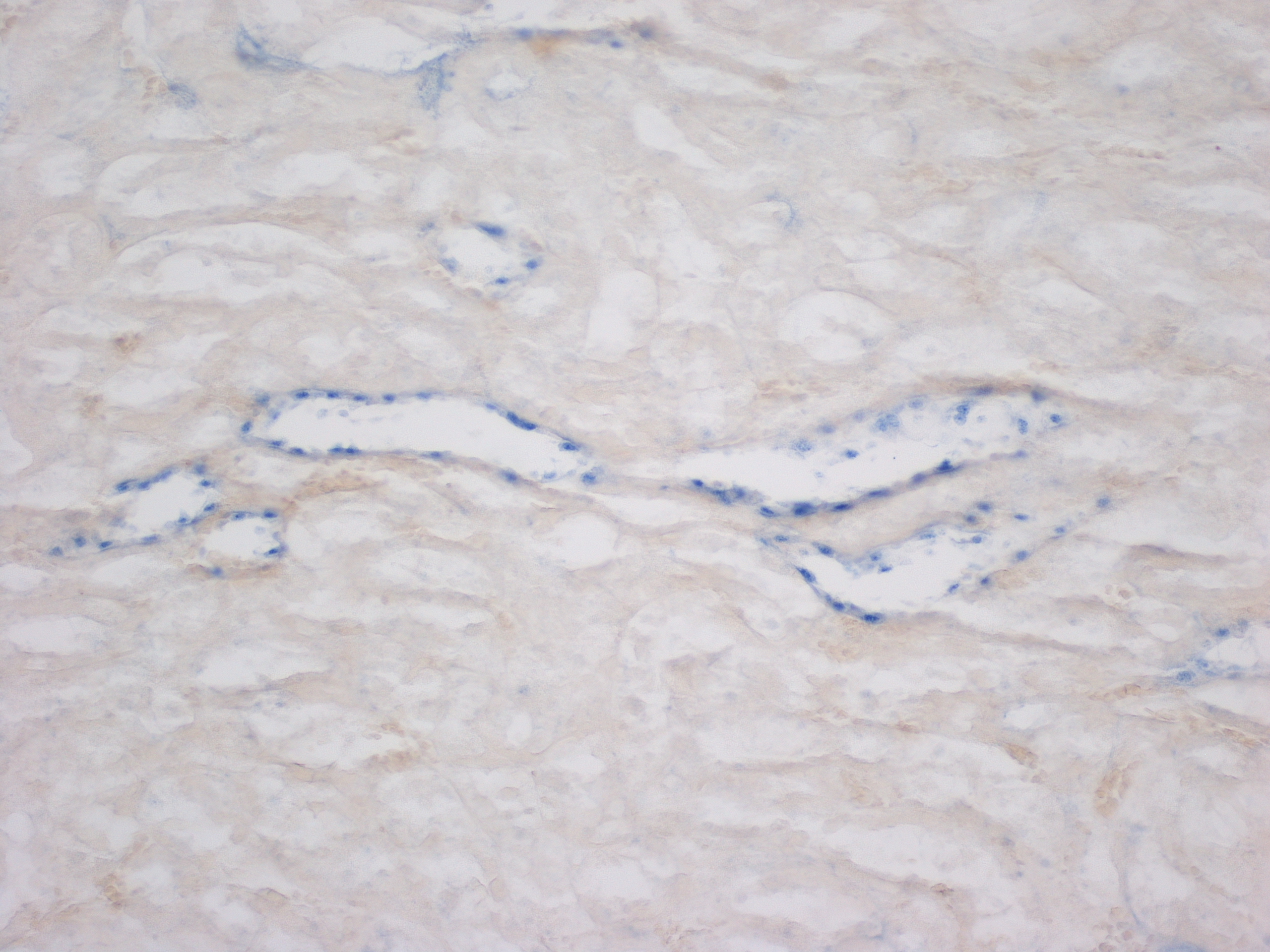

Supplement: Additional file 2 — In situ hybridization analysis of AQP6 mRNA expression in rat kidney. The probe detects intercalated cells of the collecting ducts in rat kidney. [file 1472-6793-9-18-S2.TIFF]

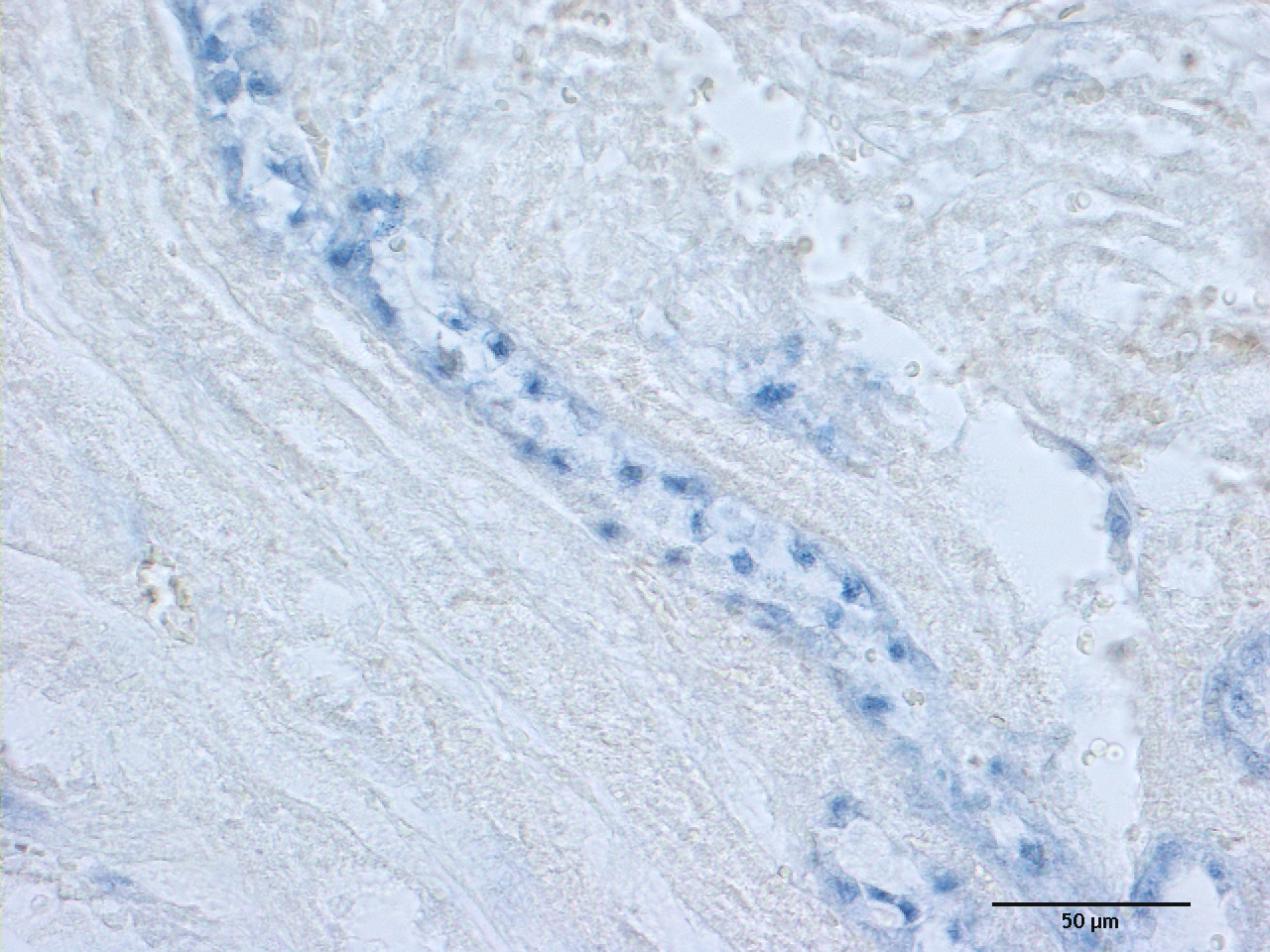

Supplement: Additional file 3 — In situ hybridization analysis of AQP6 mRNA expression in rat kidney. The probe detects intercalated cells of the collecting ducts in rat kidney. [file 1472-6793-9-18-S3.TIFF]

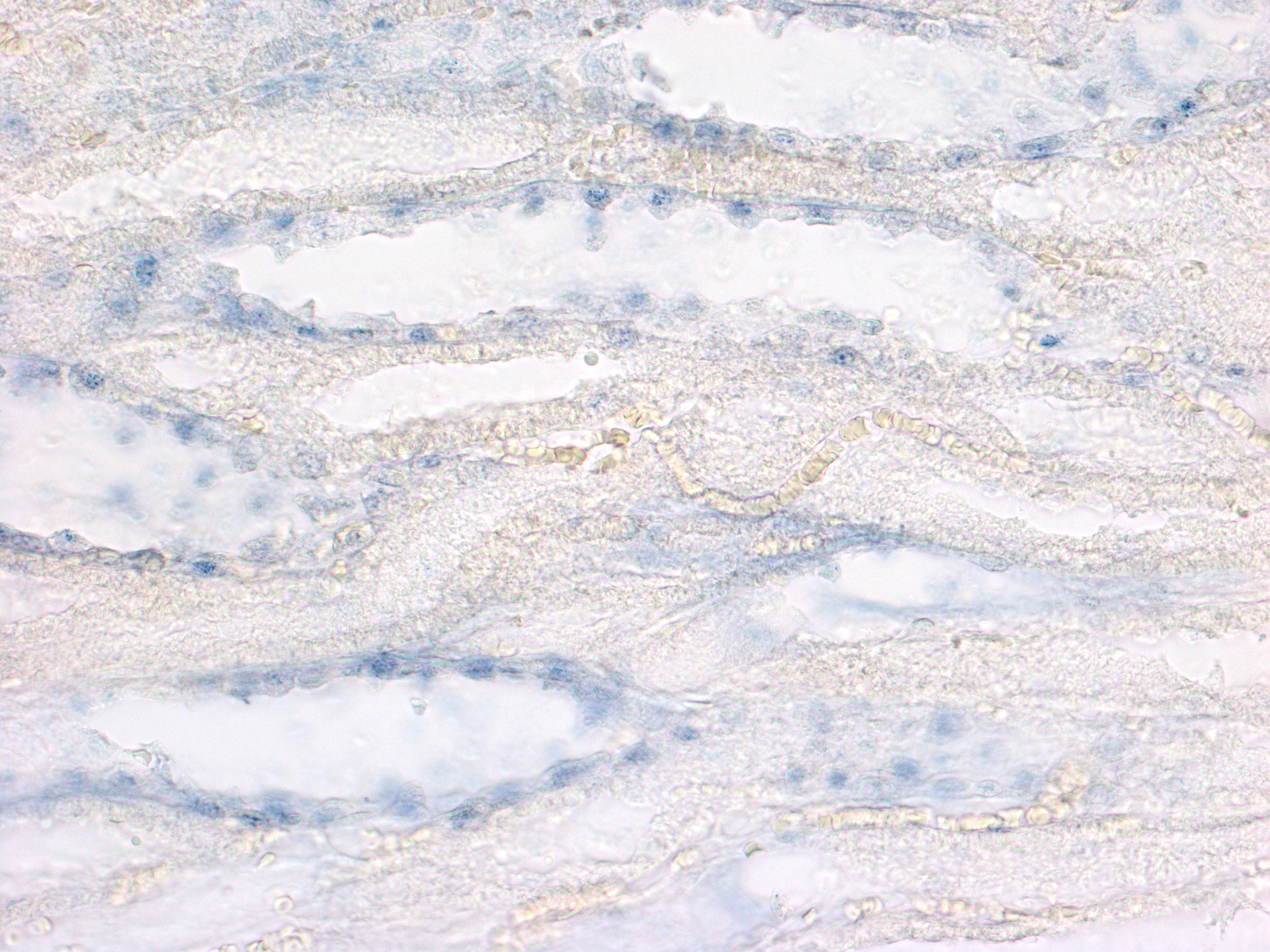

Supplement: Additional file 4 — In situ hybridization analysis of AQP6 mRNA expression in rat kidney. The probe detects intercalated cells of the collecting ducts in rat kidney. [file 1472-6793-9-18-S4.TIFF]
